# General Practice and Pandemic Influenza: A Framework for Planning and Comparison of Plans in Five Countries

**DOI:** 10.1371/journal.pone.0002269

**Published:** 2008-05-28

**Authors:** Mahomed S. Patel, Christine B. Phillips, Christopher Pearce, Marjan Kljakovic, Paul Dugdale, Nicholas Glasgow

**Affiliations:** College of Medicine and Health Sciences, Australian National University, Canberra, Australian Capital Territory, Australia; University of Cape Town, South Africa

## Abstract

**Background:**

Although primary health care, and in particular, general practice will be at the frontline in the response to pandemic influenza, there are no frameworks to guide systematic planning for this task or to appraise available plans for their relevance to general practice. We aimed to develop a framework that will facilitate planning for general practice, and used it to appraise pandemic plans from Australia, England, USA, New Zealand and Canada.

**Methodology/Principal Findings:**

We adapted the Haddon matrix to develop the framework, populating its cells through a multi-method study that incorporated the peer-reviewed and grey literature, interviews with general practitioners, practice nurses and senior decision-makers, and desktop simulation exercises. We used the framework to analyse 89 publicly-available jurisdictional plans at similar managerial levels in the five countries. The framework identifies four functional domains: clinical care for influenza and other needs, public health responsibilities, the internal environment and the macro-environment of general practice. No plan addressed all four domains. Most plans either ignored or were sketchy about non-influenza clinical needs, and about the contribution of general practice to public health beyond surveillance. Collaborations between general practices were addressed in few plans, and inter-relationships with the broader health system, even less frequently.

**Conclusions:**

This is the first study to provide a framework to guide general practice planning for pandemic influenza. The framework helped identify critical shortcomings in available plans. Engaging general practice effectively in planning is challenging, particularly where governance structures for primary health care are weak. We identify implications for practice and for research.

## Introduction

Primary health care, and in particular general practice, will be at the frontline in the response to pandemic influenza. Preparedness planning for this sector has lagged behind public health planning, despite evidence from SARS [1], [2] and influenza epidemics [3] of the important role played by general practice. Preparedness may be defined as the capacity to respond to a range of public health threats including natural disasters and infectious disease outbreaks, human-caused accidents and intentional attacks [4]. There is an increasing recognition of the need for an ‘all-hazards’ approach to planning that integrates acute clinical care, public health, and emergency management systems [4]. Since September 2001, the US government has invested about $5 billion to upgrade preparedness plans for emergency management systems [5], [6].

There are three challenges for pandemic planning by general practice. First, there is no systematic framework for planning this sector's response. Preparing for health threats and emergencies is an essential function of public health, but is not core business for general practice. Second, the way in which ambulatory health services will interact with each other and with the broader health system response to a pandemic is unclear. General practitioners (GPs) in Canada [7], Australia [8] and the UK [9] have expressed uncertainty about how to participate in such a response. Third, planning and implementing changes for pandemic influenza across the health system is complex. Although there is little evidence linking specific preparedness activities to effective system-wide responses to pandemic influenza [5], [6], change management theories point to a need for dynamic partnerships between general practices and other ambulatory care services, hospitals and public health departments [10]. The strength and structure of these linkages vary around the world, depending on decentralisation processes, the regulatory and legal system, and financing within health systems [11], [12]. Although general practice, or family medicine, is organised differently in different countries, there is considerable potential for transferable learning at the meso-level of management planning [11].

We aimed to develop a framework that will facilitate systematic planning for the general practice response to pandemic influenza and used it to appraise coverage of key elements in publicly available pandemic plans from Australia, England, USA, New Zealand and Canada.

## Methods

### Development of the framework

To guide planning and to appraise available plans, we adapted the Haddon Matrix, a planning tool developed in the field of injury research and intervention [13], and more recently applied to the public health response to bioterrorism, SARS [14], and pandemic influenza [15]. The matrix consists of a grid of columns of four factors (human, agent, and physical and organisational environment) impacting upon the event [15]. Pandemic influenza may be perceived as a form of injury on a mass scale and the matrix helps us understand the multi-dimensional nature of epidemics and of the associated challenges that could be expected by general practice. The framework can be readily shared with public health units and other parts of the health system, as it identifies the general practice contributions to primary health care services and to public health surveillance and control. Because all disasters are local, the matrix is flexible enough to allow a focused analysis of the smallest unit of study, such as an individual, or group of general practitioners.

The methods used to construct the cells of the modified Haddon matrix have been detailed elsewhere [16]. In brief, a team with expertise in social science, public health and general practice reviewed objectives and strategies in WHO guidelines for preparing and responding to a pandemic [17] to define the context and potential contributions of general practice. Next, we undertook a narrative review of the peer-reviewed and grey literature on pandemic influenza to identify papers that elaborated strategies relevant for general practice. A search of the peer-reviewed literature through PubMed using the terms ‘general practice’, ‘family physician’, ‘family medicine’ and various combinations of the terms ‘influenza’, ‘epidemic’, ‘preparedness’ and ‘pandemic’ yielded 24 eligible papers from 157 search results . The process of constructing the framework and populating the cells was informed by organisational theories that emphasise multilevel approaches to change from the individual to the broader health system [10], [18], and by methods for measuring [5] and improving the quality [6] of public health emergency preparedness.

### Testing the framework

We tested our framework through interviews with a purposive sample of health professionals engaged in pandemic planning. Nineteen general practitioners and practice nurses with expertise in pandemic planning were nominated by the two participating Divisions of General Practice, each of which was a national leader in disaster preparedness and response. Eight general practice policy leaders were identified by representative organisations (Australian Medical Association, Royal Australian College of General Practitioners, Australian General Practice Network). Group interviews were held with 14 state and territory public health leaders attending a national pandemic preparedness meeting. We held two workshops, attended by representatives of state and territory health services, Commonwealth policymakers, non-government organisations, and general practice organisations. In addition, we conducted two focus groups of GPs and nurses working in aged care in two cities. Finally, we undertook four desktop exercises [19] attended by 25 GPs, 11 practice nurses and 10 administrative staff.

### Assessment of general practice coverage in pandemic plans

The five countries in this study had national response plans. Contextualised detail about health-sector responses is contained in plans at the level of administrative decentralisation where decisions are made about patient-service groupings including general practice. In practice, this level was the state or provincial health departments in Federal systems where those jurisdictions have responsibility for health service management and planning (USA, Canada, and Australia). In England, the managerial level for health services is located at the Primary Care Trust (PCT), while in New Zealand it occurs at the level of the District Health Board. Although these are not identical loci of health service governance, they were sufficiently similar in the planning aims for comparisons to be drawn.

Plans were obtained from websites of health departments of states or provinces (USA, Australia, Canada), District Health Boards (New Zealand) and PCTs (England) (Figure S1). For New Zealand and England, publicly available records of Board Meetings were also examined. Consumer information and isolated sub plans (e.g. for infection control) were excluded. Plans for 95 jurisdictions were identified; six were excluded as they addressed isolated aspects such as only the distribution of medications, or communication with the public, leaving 89 plans suitable for analysis.

Of the five countries, Canada exhibits the most variation between provinces in health system coordination. We examined the websites of Canada's 84 provincial regional health authorities (RHAS, 14 plans identified) and Ontario's 36 public health units (26 plans identified) and 14 Local Health Integration Networks (no pandemic plans identified). We excluded the RHA and public health unit plans from inter-country quantitative analysis, as their level of devolution and/or responsibilities for health management differed from those examined in the other four countries, but have included descriptive details from some of the RHA plans where they illustrate innovative approaches.

All plans were examined by two clinicians, and searched for the following terms: primary care, primary health, ambulatory, general practice, general practitioner, GP, family practice, family physician. The roles of general practice/family practice in the plans were assessed across the four domains of general practice identified in the first part of this project. No attempt was made to quantify the extent of coverage of general practice in the plans as this rarely extended beyond a few sentences. Where there was detailed coverage of an issue, we analysed the text and the health system context.

The study was approved by the Australian National University Human Research Ethics Committee and the National Research and Evaluation Ethics Committee of the Royal Australian College of General Practitioners. Written informed consent was obtained from participants.

## Results

A conceptual framework of the general practice response to pandemic influenza is shown in Table 1.

**Table 1:** Conceptual framework of the general practice response to pandemic influenza

Domain of practice	Challenges anticipated during a pandemic	Responses to be addressed in the general practice pandemic plan
*Clinical services*	Surge in demand for primary care services for influenza	Ways to enhance surge capacity for responding to influenza
	Sustaining other urgent or essential primary care services	Maintaining other urgent and essential clinical services
*Public health responsibilities*	Effective surveillance of acute respiratory infections	Contributing data and specimens for clinical and laboratory-based surveillance
	Implementing influenza control measures	Assisting public health units with contact tracing and monitoring people in isolation or quarantine, dispensing antiviral medications and the pandemic influenza vaccine
*Internal environment of the general practice unit*	*The physical environment:*	
	Minimising the risk of spread of influenza in the practice setting	Structuring clinical facilities and stockpiling personal protective equipment to enable effective infection control
	*Organisational environment*	
	Reliable delivery of medications and essential equipment to the practice	Ensuring emergency access to essential drugs, vaccines and equipment
	Ongoing communications with patients and the health system	Strengthening capacity of communication systems
	Organisational arrangements to sustain efficient and effective services	Customising business continuity plans to the local context
		Training in use of clinical decision-making tools and conducting simulation exercises
*Macro-environment of general practice (the health system context)*	Overall organisation of the health system that will facilitate or impede effective functioning of general practice	Integrated planning across the health system, e.g. with other general practices and ambulatory care services, public health units and hospitals.
		Appropriate legislation, e.g. to address professional accreditation, indemnity, and ethical concerns
		Financing mechanisms for general practice

The framework identifies four domains of practice: clinical services, public health responsibilities of general practice, internal (physical and organisational) environment of the general practice unit, and the macro-environment of general practice. In each domain, we list the key challenges to be anticipated by general practice during an influenza pandemic, and the type of responses that need to be addressed in the plan.

Table 2 summarises the organisational levels in the five countries, the proportion of jurisdictions with accessible pandemic plans, and coverage of general practice in these plans. While almost all plans from US jurisdictions were accessible, three quarters of Australian states/territories and one third of New Zealand's District Health Boards had accessible plans. Only 13% (20/152) of England's PCTs had pandemic plans available in the public domain.

**Table 2:** Summary of organisational levels in the five countries, the proportion of jurisdictions with accessible pandemic plans, and coverage of primary health care in the plans

	USA	England	Canada	Australia	New Zealand	Total
Organisational level coordinating health system pandemic response	State	Primary Care Trust	Province/Territory	State/Territory	District Health Board	
Number of jurisdictions/organisations oversighting pandemic planning	51#	152	13	8	21	245
Number of publicly-available pandemic plans * (% of jurisdictions/organisations)	49 (96)	20 (13)	8 (62)	6 (75)	6 (29)	89 (36)
Number of pandemic plans which make reference to primary health care or ambulatory care (% of available plans)	37 (76)	20 (100)	8 (100)	5 (83)	6 (100)	76 (85)

# Includes District of Columbia

* The jurisdictions are shown in Figure S1

Figure S1 shows the jurisdictions and health management systems whose plans were included in this study; they comprise 49 jurisdictions from the USA, 20 from England, 8 from Canada, and 6 each from Australia and New Zealand.

Table 3 shows the number and rates of coverage of each of the four domains of the general practice response in jurisdictional plans of the five countries. The domain covered most frequently was influenza-related clinical care (in all plans from England and Canada). Overall less than half the plans mentioned non-influenza clinical care, with the exception being England, where 90% of PCT plans mentioned non-influenza clinical care. Public health surveillance was addressed in all plans from Canada and New Zealand and infection control in general practice in almost all plans from England and Canada. Functional linkages of general practice with other parts of the health system were addressed in almost all the English plans, but a smaller proportion of other plans.

**Table 3:** Number and rates of coverage of each of the four domains of general practice in the jurisdictional pandemic plans of the five countries

Coverage of general practice response domains	Number of plans addressing domains (%)					
	USA *n = 49*	England *n = 20*	Canada *n = 8*	Australia *n = 6*	New Zealand *n = 6*	Total *n = 89*
**Clinical care**						
Influenza-related care	20 (41)	20 (100)	8 (100)	4 (66)	4 (66)	56 (63)
Non influenza related care	14 (29)	18 (90)	3 (38)	3 (50)	2 (33)	40 (45)
**Public health**						
Surveillance	26 (53)	6 (33)	8 (100)	5 (83)	6 (100)	51 (57)
Immunisation^1^	15 (31)	9 (45)	3 (38)	4 (66)	2 (33)	33 (37)
**Internal environment**						
Infection control	19 (39)	18 (90)	8 (100)	4 (66)	4 (66)	53 (60)
**Macro-environment**						
Linkages between health services	10 (20)	19 (95)	3 (38)	3 (50)	4 (66)	39 (44)

1 Includes immunisation against seasonal influenza, pneumococcal infection as well against pandemic influenza

### Clinical care

#### Essential planning elements

This domain includes two sets of clinical care needs. The first, prevention and treatment of influenza, includes care for the surge in patients with acute respiratory illness, and for people at high risk of exposure to, or complications from, influenza. These aspects are discussed extensively in the literature [20]–[23]. Most people with influenza can be managed in the community, protecting hospitals by delaying or avoiding admission and facilitating early discharge.

The second clinical care need is for non-influenza-related care. General practitioners provide most chronic disease care, though there is inter-country variation in their capacities to do this efficiently [24], [25]. While activities like cervical screening may cease in a pandemic, chronic illnesses like diabetes or cardiac disease will still need management. Some acute care usually undertaken in hospitals, like acute asthma or injuries, may be transferred to the community. In an earlier paper, we advanced a range of models of practice to balance clinical services for influenza and non-influenza care [16].

In the recovery phase, the clinical needs of patients are for psychological care and chronic illness management. If the pandemic occurs in waves, as in 1918–19, recovery activities may need to be tempered by preparations for the next wave.

#### Coverage of essential elements in plans

All Canadian and English plans outlined a role for general practice in clinical care for influenza. While only 41% of plans from the USA addressed clinical care for influenza by primary care practitioners (Table 3), every US plan included guidelines on influenza management by hospital physicians. Some plans articulated a surge in demand for influenza care as a threat to general practice's survival, and proposed assessment and treatment clinics as a way of protecting them [26], [27]. In other plans [28]–[30] the response to a surge was to support general practices to become more resilient by collaborating and changing their work practices. In two US state plans, the failure of the ambulatory care sector in the face of a surge was assumed. The planning challenge became to find ways to redeploy workers into other health care sectors [31], [32].

Most plans were sketchy on systems to maintain non-influenza-related clinical care, with the exception of some PCT plans, which included activities like triage, extended prescribing, identifying deferrable reasons for presentation, and management of more acute problems to protect hospitals [29], [33]–[36]. The main non-influenza clinical area was mental health care, mentioned in six plans from the USA [37]–[42] (reflecting a focus in the national plan [43]) and one Canadian plan [44]. Coverage of the needs of vulnerable populations– the elderly, homeless, prisoners and the psychologically unwell – was most detailed in plans from Canada and England.

### Public health responsibilities of general practice

#### Essential planning elements

This domain includes surveillance of influenza-like illness and influenza virology, and control of influenza in the general practice and the community. Surveillance includes early diagnosis and notification, and specimen collection to confirm clinical diagnosis and to monitor viral characteristics and resistance to antiviral drugs. GPs and private specialists are currently central to surveillance activities [45]–[48]. In the early stages of the pandemic, it is likely that public health authorities will undertake contact tracing to facilitate containment, but their capacity to sustain this approach as the epidemic continues will be limited. General practice may then be expected to include contact tracing, and monitoring and support of people in quarantine or home isolation. Other responsibilities may include prescribing and dispensing antiviral drugs and participating in mass immunisations against the pandemic strain of the virus.

#### Coverage of essential elements in plans

Surveillance in general practice was mentioned in 53% of US plans and in only 33% of English plans, in all Canadian and New Zealand plans, and all but one Australian plan (Table 3). The low rates of coverage of surveillance in PCT plans are not in accord with the UK plan which imputes to general practice a role in surveillance, and recommends that PCTs operationalise this recommendation [49]. The College of Family Physicians in Canada is a partner in FluWatch, recruiting sentinel physicians to undertake surveillance, so this role is well understood within the Canadian health sector.

The role of general practice in contact tracing, in monitoring people in home isolation, and in distributing antiviral drugs is unclear in most plans. Home care by GPs for people in quarantine is mentioned in two US Plans [50], [51], and one English plan [36], though the recently released guidelines for PCTs anticipate a role for general practices in home care [52]. In all country plans, dispensing antiviral medications was generally performed by public health units. Only 22% of PCT plans and 40% of US plans mention a role for primary care in dispensing antiviral medications. None of the Canadian plans, and only one NZ and two Australian state plans, mentioned antiviral dispensing by primary care. The only plan to set out contingencies when decisions about dispensing may change was one Canadian RHA plan [27]. Although immunisation was mentioned most frequently after surveillance as a public health activity by general practices, in most plans the immunisations were against pneumococcal disease and seasonal influenza, but not mass immunisations against pandemic influenza.

### Internal environment of general practice

#### Essential planning elements

This domain includes the physical environment of the general practice and its practice-level organisation. The risk of transmission of infections within the surgery could be minimised through separate waiting rooms and entrances, triage and personal protective equipment and hand-washing facilities. Hogg has outlined infections control procedures in the practice and the associated financial costs [53]. Some general practices (for example, those with small waiting rooms, or only one consulting room) may be deemed too much of a transmission risk to continue providing face-to-face services.

The practice needs to develop strategies to maintain reliable and efficient access to essential drugs and equipment and influenza and pneumococcal vaccines. It also needs to strengthen the capacity of its communication technologies with patients and the broader health system, including telephones, faxes, internet, work-from-home technologies for staff, compatible software for sharing electronic medical records, and recall and reminder systems for patients.

Preparation at the organisational level relates mainly to business continuity plans. These plans should include leadership delegations, staffing contingencies, safe and flexible working hours and family care plans for staff, criteria for considering clinic closure, recruiting and training ancillary staff, early psycho-social support, support for making difficult clinical decisions, record keeping to ensure accountability for actions and ‘inactions’, use of antiviral medications, and plans for simulation exercises to complement training, and to evaluate and refine local practice plans. Tools [54], [55] and desktop simulation exercises [19] are available to help GPs plan for continuity.

#### Coverage of essential elements in plans

Infection control strategies were well covered in plans from Canada and England, but were mentioned in only 39% of US plans (Table 3). None of the plans provided an inventory of fixed features, such as size and layout of waiting room, or a single entrance, which could compromise infection control.

Business continuity was a focus of the English plans, which frequently referenced resources available on the UK Resilience website [56]. This aspect of preparedness was enhanced after the Exercise Winter Willow simulation in February 2007, and new PCT guidelines addressing workforce planning [52]. Some PCT plans addressed the need for general practice resilience in the face of workforce sicknesses [33], increased aggression from patients, and threatened loss of capacity in single doctor practices [57]. Few plans from other countries discussed business continuity for primary care in such detail. This may be because such issues are felt to be outside the normal purview of state or provinces, and to be the responsibilities of the businesses themselves or corporate interests.

### Macro-environment of general practice

#### Essential planning elements

This domain includes the overall organisation of, and interactions with, the health system that will facilitate or impede effective functioning of general practice services during a pandemic, including adaptation of relevant regulatory and financing systems.

The health system requires a plan that adopts the ‘all-hazards approach’ and integrates roles, responsibilities and actions for acute clinical care, public health, and emergency management systems [4]. This calls for coordination across general practices and other ambulatory care services to ensure primary health care needs within the community are effectively monitored and addressed; with hospitals to avoid/delay hospitalisation and facilitate early discharge; and with public health units to share responsibilities for contact tracing, monitoring and treating people in home isolation or quarantine, dispensing of anti-viral medications, and participation in mass immunisations against pandemic strains of the virus (when these become available).

Neighbouring general practices and other ambulatory care services will need local leadership with strategic approaches to collaborate and maintain services through a pandemic. England's PCTs and New Zealand's Primary Health Organisations (PHOs) represent two ways of linking general practices under the governance of regional boards. These networks are consolidated by financial relationships between the PCT or the PHO and general practices. The links between Australia's Divisions of General Practices and GPs are purely voluntary. In the USA, managed care systems function as another way of linking ambulatory and hospital services. Communication infrastructure between Canada's family practitioners, 25% of whom are solo practitioners [58], is still being developed, as is the incorporation of general practice into Canada's Pan-Canadian Public Health Network [59].

The regulatory environment includes accreditation of retired medical practitioners and allied health professionals, laws and regulations which support or hinder the flow of qualified personnel across a jurisdiction's health facilities [48], and ensuring an appropriate medicolegal framework to support clinical decisions on prioritising medical care during a pandemic, for example, modifying clinical standards, deferring treatment, and restricting access to certain treatments.

Funding mechanisms for general practice may impact upon the capacity to provide extra services. In countries with fee-for-service payment systems, general practices may profit from a surge in attendances, but may equally run into business difficulties if they are short-staffed for prolonged periods. GPs funded through a capitated system may have more freedom to alter their practice to provide different service mixes.

In the post-event phase, patients and GPs may require support for psychological recovery. It may be necessary to provide some formal relief through a system of locum GPs from areas less affected by the pandemic. Organisational partnerships at this stage may need to be with social services and mental health support services.

#### Coverage of essential elements in plans

Countries with mechanisms for linking general practices with other sectors were more likely to address networking in their plans. Ninety five per cent of English plans addressed systems to support collaboration between general practices (Table 3). These plans addressed buddy systems, practice networks, and contingency plans for communities of practice. Four of the six New Zealand plans also addressed collaboration, though only one in significant detail; this plan outlined a distinction between key practices, and other practices which might decide to partner one another [55]. Of the three Canadian provincial plans that addressed collaboration, the most comprehensive was from Quebec, which identified a need to bridge the gap between salaried practitioners and independent physicians. The plan of the Montreal Regional Authority [60] operationalises this by setting up a system of active and sustained outreach by the public health department to independent physicians.

The absence of plans for networking between general practice and public health is most marked in the USA. With the exception of Louisiana [61], US plans which mentioned networking did so in one line, generally advocating partnership between private and public services without indicating how this might occur. Louisiana's strategic approach built a participatory structure for rural practitioners through a partnership between the state public health department and the Bureau of Primary Rural Health Care.

The Canadian national pandemic plan [62] is framed around a set of ethical precepts incorporated into pandemic planning at the provincial and regional health level. The UK has recently released an ethical framework for policy and planning, though this has not yet been incorporated into planning documents [63]. The regulatory framework most mentioned was in relation to credentialing for retired GPs and other volunteers [33], [64], [65], and less frequently, indemnity [36]. Although most plans include coverage of the relevant public health legislation, no country's plan included an inventory of legislation relevant to general practice that might need to be amended.

Only one plan [66] and the PCT guidelines [52], canvas the potential of recompense for financial loss to a general practice. The only country in which the planning level coincided with the level that made decisions about funding of health care was Canada. One regional health authority plan provided an outline of specific issues likely to affect physicians, and raised the possibility of reviewing funding mechanisms in a pandemic [67]. There appear to be no ancillary plans addressing principles of altered funding for private physicians in a pandemic.

## Discussion

This is the first study to provide a framework that brings together multiple functions, structural relationships and the responsiveness of general practice to prepare for pandemic influenza. The framework provides clarity of purpose and a structure to guide planning through four functional domains: clinical care, public health responsibilities, and the internal and macro environments of general practice. The domains have been structured as integral components of a complex system that can respond to uncertainty [68] and be adapted for a given local setting and health system context.

We draw three conclusions regarding general practice from our analysis. First, none of the 89 jurisdictional plans addressed all domains of the general practice response during a pandemic. Second, while many aspects of the first three domains are included in plans for general practice, there are critical gaps and inconsistencies in the fourth domain (macro-environment) that render some elements of the jurisdictional plan ungrounded or unrealistic. Third, few plans addressed the broader ambulatory care context, including the need to engage private specialists and other allied health professionals [48].

Planning and implementing change across the health system is complex. Targeting individual sectors for change (e.g. public health departments, hospitals or general practices) without securing reciprocal changes and strengthening inter-relationships across the health system, is unlikely to succeed [10], [18]. Planners must consider how connectivity across the health system might be strengthened to enable optimal use of general practice resources for planning [68]. While this may be challenging, particularly in countries with weak governance structures for primary health care, omitting general practice input into the planning process may be considered unethical [69] and counterproductive.

*Limitations of the study:* Our findings are exploratory rather than definitive, and indicate directions for further planning and research. Like any new tool, the framework and its application in a given context needs testing and refinement through simulation exercises targeting ambulatory care services as well as the broader health system.

Planning is an evolving activity that reflects a ‘map’ rather than a ‘destination’, and our findings provide a snapshot of the plans accessible in late 2007. The scope and content of the plans will change over time, as seen in two countries that adjusted their plans after simulation exercises, Exercise Cumpston in Australia [70] and Winter Willow in the UK [71]. Interestingly, the former identified specific weaknesses in the involvement of the primary health care sector and made recommendations to better integrate primary health care providers into planning at the national and jurisdictional levels [70]. National and sub-national pandemic plans may be intended to provide a strategic focus and not to elaborate on operational activities; it is possible the latter may have been addressed, but were not accessible at the time of our study.

Another potential limitation of our study is that the gaps we identified in many plans were grounded in theories about the ways to enhance the quality and outcomes of clinical care [10], [18] or of public health preparedness planning [6]. The science of preparedness planning is still maturing [4]–[6] and there is relatively little systematic evidence for linking specific preparedness structures to the ability to implement efficient and effective responses [5], [6].

Two important limitations to the implementation of preparedness activities are uncertainties in knowing how much preparedness is enough [5] and in having a measurable assessment of the outcomes of preparedness activities. It may be more meaningful to perceive of the activities as a ‘preparedness production system’ in which a variety of processes and activities have been completed to prepare for an optimal response [6]. We are unable to comment on the extent to which these preparedness plans have been implemented, except in the case of those jurisdictions which have held pandemic exercises [70], [71]. General practice response is rarely tested in pandemic exercises, which tend to focus on hospital and public health responses. A notable exception is Operation Sparrowhawk in Singapore, where the feasibility of general practice influenza clinics was tested [72]

The Haddon matrix is not a final check-list for preparedness planning but a problem-solving tool used as a starting framework for planning. The contents of each cell of the matrix help identify a particular problem or challenge that needs to be addressed. We recognise that the challenges will be neither static over time, nor uniform across general practices; responses will have to be modified in the context of the general practice setting as the pandemic evolves and as other parts of health system, particularly hospitals and public health units respond to the epidemic.

*Implications of our study for primary health care in developing countries:* Endemic and epidemic infectious diseases inflict high levels of morbidity and mortality in developing countries because of a combination of poor living conditions, effects of multiple concurrent illnesses particularly in children, fragile national health systems, overburdened and overstressed health workers, and negative work environments [73]. Although our study targeted general practice in developed countries, the conceptual framework we developed (Table 1) can be used by primary health care services in developing countries to deconstruct the multi-dimensional challenges posed by pandemic influenza. Identifying possible solutions and apportioning responsibilities across components of the health system is more complex. Operational guidelines have been developed for the detection and rapid containment of a potentially pandemic strain of influenza to the epicentre of the outbreak [74], for example, if this were to occur in a South East Asian country. However, because of the immense global implications of such an event, this intensive strategy will need to be supported by extraordinary resources from the global community, an action not sustainable once the pandemic strain spreads beyond the initial epicentre.

In an analysis of pandemic influenza plans in Asia-Pacific countries in 2006, Coker found that although all countries recognised the importance of pandemic planning, operational responsibility particularly at the local level, remained unclear; most plans relied on specialised flu hospitals, while few developed the possibility of caring for patients at home [75]. (The study made no reference to primary health care or the private practice sector). In his analysis of public health emergencies in developing countries, Quarantelli identified relatively poor adaptive capabilities to be the key barrier to effective responses at the central and local levels [76]. Possible reasons included poorer public health infrastructures and human and financial resources, organisational structures that functioned mainly in a top-down manner with a strong emphasis on structures more than functions, and lack of planning initiatives the further away one moved from central level [76].

Many poor countries already have a health crisis, and need massive international investments, including mobilisation and strengthening of human resources to build sustainable health systems, strong leadership and political commitment [73]. In the face of the pandemic threat, primary health care in developing countries will need resources to develop a suite of policies, including: clarification of what essential primary health care will continue through a pandemic, developing health workforce plans that may entail diverting clinicians from other areas of the health workforce, establishing non-hierarchical links between primary health care, hospitals and public health, and injecting funds into hospital and primary care preparedness simultaneously.

### Enhancing the role of general practice in pandemic planning

It may be argued that the absence of general practice elements from pandemic plans is not problematic, that it is outside the responsibility of public health departments that do not have a governance role for general practice. We argue instead that the general practice sector, which is characterised by loose networks between ambulatory care services, and often lacks the appropriate organisational structure and mandate, cannot spearhead many elements of planning for primary care. This calls for actions by health departments as well as by general practices.

#### Actions by health departments

Ensuring that the community receives appropriate health care during public health emergencies is a government responsibility. Consequently, health departments must emphasise in national and sub-national plans, the critical need for all levels of the health system to integrate the general practice sector in the planning process. This should include appropriate general practice representation in high level planning and decision-making committees, in incident-command-control structures and in the management of community-based specialised clinics such as ‘fever clinics’ or ‘community information and assessment centres’.

Good planning must focus on the planning process rather than the production of a written document [76]. The process includes collaborative activities such as meetings, drills, exercises, simulations, developing techniques for training, knowledge transfer, identifying and obtaining resource materials, and continually updating materials and strategies. These planning activities are important not only because they inform, but because they also foster collaborative learning and problem-solving, and generate an atmosphere of mutual trust and solidarity among people who will be affected by a pandemic and whose collaboration will be essential in the response.

The willing general practitioner sector [7], [8] is an essential resource for extending the surge capacity of health departments. Health departments should harness and support interactions and networking among general practices, and between them and ambulatory health care providers, hospitals and public health units. The role of general practice in contact tracing, monitoring and treating people in home isolation or quarantine, dispensing antiviral drugs and participating in mass vaccinations - omitted in most plans - needs to be clarified. In addition, health departments should modify or adopt where appropriate, legislation and financing mechanisms to enable general practices to function optimally during the pandemic.

#### Action to support planning by general practice

While the diversity of the general practice sector means that there will not be guidelines to cover all scenarios and contexts, a coherent approach would enable multi-actor accountability and more efficient, contextual planning by jurisdictions. The guidelines for PCTs [52] are an example of such an approach, designed for a particular health system. They could act as a useful point of departure for planning integrated general practice plans by other health systems.

There is a need for a system of sharing innovations and exemplary solutions to challenges for pandemic planning by general practice, analogous to those targeting mainly hospitals and public health departments [77]. Given the diversity in organisation of general practice systems, a web presence comparing exemplary approaches from different health systems would be a useful resource for planners.

### Implications for research

An important challenge will be ensuring collaboration and coordination across the health sector during a pandemic. Research is needed to identify the prevailing barriers and facilitators to effective collaboration across the health sector, how these may change under the stressor of a pandemic, and how this information could be used to optimise the response.

The regulatory environment is founded on a set of ethical principles, often unarticulated. Since there is likely to be some dispute between utilitarian philosophical approaches used in public health and deontological or virtue ethical approaches used in clinical medicine [78], there is a need for some preparatory work with general practitioners clarifying ethics of clinical behaviour, restriction of liberty under quarantine orders, and resource allocation and distribution.

In an established pandemic, it is likely that there will be shortfalls in the GP workforce, due to illness among GPs, caring duties or closure of small practices. Non-hospital clinical specialists, retired general practitioners, allied health professionals and medical students could be trained to fill the gap in services. Research is needed to define the clinical work that can be done by other health personnel in general practice, eligibility criteria and accreditation processes for this cadre of workers, and optimal training processes.

### Conclusions

All public health problems have a clinical dimension, and all clinical problems have a public health dimension. At present, the plans in the five countries provide more detail on the public health dimension of the pandemic. There are intercountry differences in the emphases provided to different domains of the general practice response. Some of this reflects the emphasis on particular elements contained within the relevant national plan. Some of the differences are due to the ways in which general practice is structured in a country, and the strengths of its linkages to other components of the health sector. There is an urgent need to incorporate general practice and the broader primary care sector into pandemic planning activities, and to undertake the preparedness activities that would make this sector, which provides the majority of health care work, a true partner in pandemic response.

## Supporting Information

Figure S1 Jurisdictions or health management organizations whose plans were included in the study.
(0.04 MB DOC)
