# Supplementary material for: General Practice and Pandemic Influenza: A Framework for Planning and Comparison of Plans in Five Countries
Source: PLoS One. 2008 May 28;3(5):e2269. doi: 10.1371/journal.pone.0002269 (PMC2386973; doi:10.1371/journal.pone.0002269)
Supplement: Figure S1 — Jurisdictions or health management organizations whose plans were included in the study. (0.04 MB DOC) [file pone.0002269.s001.doc]

Figure S1: Jurisdictions or health management organisations whose plans were included in the study

| USA | Alabama Department of Public Health.  State of Alaska, Division of Public Health.  Arizona Department of Health Services.  Arkansas Department of Health and Human Services..  California Department of Health Services.  Colorado Department of Health and Environment  Connecticut Department of Public Health.  State of Delaware Department of Health and Social Services  District of Colombia Department of Health.  Florida Department of Health.  Georgia Department of Human Resources  Hawaii State Department of Health.  Idaho Department of Health and Welfare.  State of Illinois.  Indiana State Department of Health  Iowa Department of Public Health.  Kansas Department of Health and Environment  State of Kentucky Louisiana Department of Health and Hospitals..  Maine Bureau of Health.  Maryland Department of Health and Mental Hygiene  Massachusetts Department of Public Health.  Michigan Department of Community Health.  Minnesota Department of Health.  Missouri Department of Health and Senior Services  Montana Department of Public Health and Human Services  Nebraska Health and Human Services System.  Nevada State Health Division and Department of Health and Human Services.  New Jersey Department of Health and Senior Services.  New Mexico Department of Health  New York State Department of Health  North Carolina Division of Public Health  North Dakota Department of Health.  Ohio Department of Health.  Oklahoma State Department of Health  Oregon Department of Human Services  Pennyslvania Department of Health.  Rhode Island Department of Health.  South Carolina  South Dakota Department of Health.  Tennessee Department of Health  Texas Department of State Health Services  Utah Department of Health..  Vermont Department of Health.  Virginia Department of Health..  Washington State Department of Health.  West Virginia State.  Wisconsin State  Wyoming Department of Health. |
| --- | --- |
| New Zealand | Counties Manukau District Health Board  Bay of Plenty District Health Board and  Lakes District Health Board ^1^  Mid Central District Health Board  Otago District Health Board and  Southland District Health Board ^1^ |
| Canada | Ontario Ministry of Health and Long-Term Care  British Columbia Ministry of Health  Government of Nova Scotia  Prince Edward Island Department of Health.  Government of Quebec  Government of the Northwest Territories  Government of New Brunswick  Government of Newfoundland and Labrador |
| England | Bath & North East Somerset Primary Care Trust.  Sutton & Merton Primary Care Trust.  Berkshire East Teaching Primary Care Trust  Camden Primary Care Trust  Brent Teaching Primary Care Trust.  Surrey Primary Care Trust  South Tyneside Primary Care Trust.  Sunderland Teaching Primary Care Trust  Greenwich Teaching Primary Care Trust  Barking & Dagenham Primary Care Trust.  Leicestershire County & Rutland Primary Care Trust  Ashton, Leigh & Wigan Primary Care Trust  Hillingdon Primary Care Trust  Brighton & Hove City Primary Care Trust  South West Hampshire Primary Care Trusts (Eastleigh & Test Valley South PCT and New Forest PCT).  North Devon Primary Care Trust.  Westminster Primary Care Trust.  Wiltshire Primary Care Trust  Dorset Primary Care Trust  Redbridge Primary Care Trust |
| Australia | New South Wales Department of Health  Western Australia Department of Health  Victoria Department of Human Services  Tasmania Department of Health and Human Services  Queensland Health  South Australia Department of Health |

^1^ Shared plan
